# Diaqua­bis(ethyl­enediamine-κ^2^ *N*,*N*′)copper(II) bis­(4-phenyl­benzoate) 2.66-hydrate

**DOI:** 10.1107/S1600536810016223

**Published:** 2010-05-08

**Authors:** José A. Fernandes, Ana I. Ramos, Patrícia Silva, Susana S. Braga, Paulo Ribeiro-Claro, João Rocha, Filipe A. Almeida Paz

**Affiliations:** a Department of Chemistry, University of Aveiro, CICECO, 3810-193 Aveiro, Portugal

## Abstract

In the title complex, [Cu(C_2_H_8_N_2_)_2_(H_2_O)_2_](C_13_H_9_O_2_)_2_·2.66H_2_O, the Cu^II^ centre (located at an inversion centre) is coordinated by two bidentate ethyl­enediamine (en) ligands and two water O atoms in a typical Jahn–Teller distorted octahedral geometry. The amino groups and the water mol­ecules are disordered over two distinct crystallographic positions with occupancies of 1/3 and 2/3. In the crystal, the cations and anions are disposed in alternating layers. One of the water mol­ecules of crystallization is disordered and the other has a fractional occupation. In the 2/3 occupancy component, water mol­ecules are organized into a chain composed of hexa­meric units inter­connected by carboxyl­ate bridges.

## Related literature

For general background to reactions based on the copper cation, see: Graham *et al.* (2000 ▶); Majumder *et al.* (2006 ▶); Rao *et al.* (2004 ▶); Zhao *et al.* (2009 ▶). For examples of framework-type structures of hybrid materials comprising carboxyl­ate anions, see: Eddaoudi *et al.* (2001 ▶). For general background to crystal engineering approaches from our research group, see: Paz & Khimyak *et al.* (2002 ▶); Paz & Bond *et al.* (2002 ▶); Paz & Klinowski (2003 ▶); Paz *et al.* (2005 ▶); Shi *et al.* (2008 ▶). For a description of the graph-set notation for hydrogen-bonded aggregates, see: Grell *et al.* (1999 ▶).
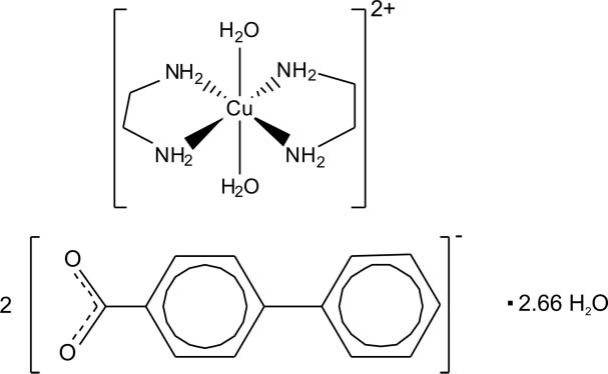


## Experimental

### 

#### Crystal data

[Cu(C_2_H_8_N_2_)_2_(H_2_O)_2_](C_13_H_9_O_2_)_2_·2.66H_2_O
*M* *_r_* = 662.20
Monoclinic, 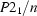

*a* = 6.1466 (6) Å
*b* = 34.984 (3) Å
*c* = 7.3101 (7) Å
β = 95.819 (4)°
*V* = 1563.8 (3) Å^3^
*Z* = 2
Mo *K*α radiation
μ = 0.76 mm^−1^
*T* = 150 K
0.13 × 0.10 × 0.06 mm

#### Data collection

Bruker X8 Kappa CCD APEXII diffractometer
Absorption correction: multi-scan (*SADABS*; Sheldrick, 1997 ▶) *T*_min_ = 0.908, *T*_max_ = 0.956
26073 measured reflections
4736 independent reflections
3908 reflections with *I* > 2σ(*I*)
*R*_int_ = 0.034

#### Refinement

*R*[*F* ^2^ > 2σ(*F* ^2^)] = 0.041
*wR*(*F* ^2^) = 0.137
*S* = 1.10
4736 reflections
259 parameters
15 restraints
H atoms treated by a mixture of independent and constrained refinement
Δρ_max_ = 0.52 e Å^−3^
Δρ_min_ = −0.66 e Å^−3^

### 

Data collection: *APEX2* (Bruker, 2006 ▶); cell refinement: *SAINT-Plus* (Bruker, 2005 ▶); data reduction: *SAINT-Plus*; program(s) used to solve structure: *SHELXTL* (Sheldrick, 2008 ▶); program(s) used to refine structure: *SHELXTL*; molecular graphics: *DIAMOND* (Brandenburg, 2006 ▶); software used to prepare material for publication: *SHELXTL*.

## Supplementary Material

Crystal structure: contains datablocks global, I. DOI: 10.1107/S1600536810016223/tk2668sup1.cif

Structure factors: contains datablocks I. DOI: 10.1107/S1600536810016223/tk2668Isup2.hkl

Additional supplementary materials:  crystallographic information; 3D view; checkCIF report

## Figures and Tables

**Table 1:** Selected bond lengths (Å)

Cu1—N1	2.019 (2)
Cu1—N1′	2.035 (4)
Cu1—N2	2.006 (2)
Cu1—N2′	2.008 (4)
Cu1—O1*W*	2.496 (4)
Cu1—O3*W*	2.605 (2)

**Table 2:** Hydrogen-bond geometry (Å, °)

*D*—H⋯*A*	*D*—H	H⋯*A*	*D*⋯*A*	*D*—H⋯*A*
N1—H1*A*⋯O2^ii^	0.92	2.13	2.986 (3)	154
N1—H1*B*⋯O2*W*^iii^	0.92	2.32	3.093 (3)	141
N2—H2*A*⋯O2^iii^	0.92	2.16	3.037 (3)	158
N1′—H1*E*⋯O2*W*^iii^	0.92	2.51	3.375 (4)	157
N1′—H1*F*⋯O1^iii^	0.92	2.20	3.087 (5)	161
N2′—H2*E*⋯O4*W*^iv^	0.92	2.33	3.181 (9)	154
N2′—H2*F*⋯O2*W*^v^	0.92	2.21	3.055 (5)	152
O1*W*—H1*M*⋯O2^iii^	0.95 (1)	1.92 (1)	2.844 (4)	163 (4)
O1*W*—H1*N*⋯O2^vi^	0.95 (1)	1.88 (1)	2.814 (4)	169 (4)
O2*W*—H2*M*⋯O2^vii^	0.95 (1)	1.73 (1)	2.668 (2)	168 (5)
O2*W*—H2*N*⋯O1^viii^	0.95 (1)	1.87 (2)	2.765 (2)	156 (5)
O3*W*—H3*M*⋯O2*W*^v^	0.95 (1)	2.01 (1)	2.932 (3)	163 (3)
O3*W*—H3*N*⋯O1^ix^	0.95 (1)	1.82 (1)	2.721 (3)	158 (3)
O4*W*—H4*M*⋯O1^x^	0.95 (1)	1.91	2.8562 (17)	178

Symmetry codes: (ii) 
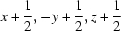
; (iii) 
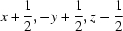
; (iv) 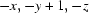; (v) 
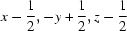
; (vi) 
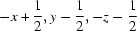
; (vii) 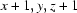; (viii) 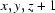; (ix) 
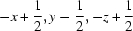
; (x) 
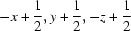
.

**Fig. 1.**
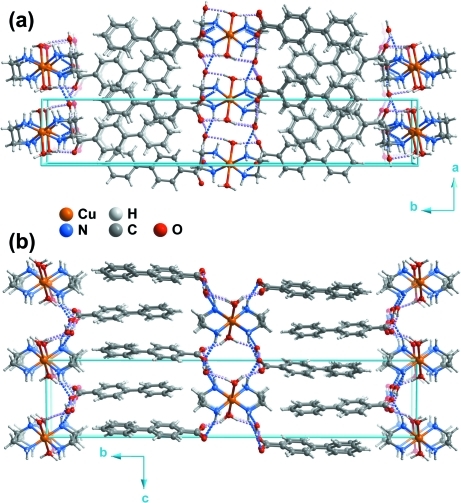
Perspective views along the (a) [001] and (b) [100] directions of the unit cell of the crystal packing of the title compound. Only the major contribution for the cation and water molecules of crystallisation are represented.

**Fig. 2.**
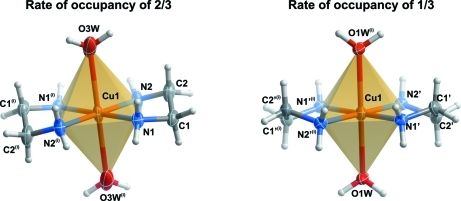
Octahedral coordination environments of the Cu centres. Symmetry transformations used to generate equivalent atoms: (i) 1-x, -y, -z.

**Fig. 3.**
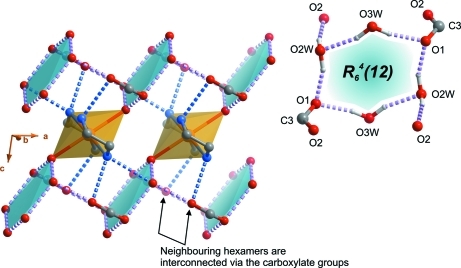
Hydrogen bonding network solely composed by the major component of the title complex. The O—H···O bonds are depicted in violet, and N—H···O bonds are depicted in blue. The organic part of the pb- ligand has been omitted for clarity. Symmetry operations used to generate equivalent atoms have been omitted for simplicity.

## supplementary crystallographic information

### Comment

Carboxylate anions are widely used in the synthesis of coordination polymers (Eddaoudi *et al.*, 2001). Our research group specialises on the design and preparation of these compounds (Paz & Khimyak *et al.*, 2002; Paz *et al.*, 2003; Paz *et al.*, 2005; Shi *et al.*, 2008) and following our previous interest in the biphenyldicarboxylate anion (bpdc^2-^) as a bridging ligand (Paz & Bond *et al.*, 2002), we have recently started to use 4-phenylbenzoate (pb^-^). Given its ability to form stable coordination complexes, copper was chosen as metal centre for our preliminary studies (Graham *et al.*, 2000; Majumder *et al.*, 2006; Rao *et al.*, 2004; Zhao *et al.*, 2009). While reacting pb^-^ with [Cu(en)_2_(H_2_O)_2_](NO_3_)_2_ we isolated as a secondary product, the title complex, (I), whose structure we wish to report now.

The asymmetric unit of (I) contains one 4-phenylbenzoate anion, 1.33 water molecules of crystallisation, and 1/2 of the [Cu(en)_2_(H_2_O)_2_]^2+^ cation; the latter is situated about a centre of inversion. The species are distributed in alternating layers along the *b* axis (Fig. 1a). An ordered hydrophobic layer is composed by the aromatic rings from pb^-^ and occupies the unit cell regions located between *ca.* 0.1 < *b* < 0.4 and 0.6 < *b* < 0.9 (Fig. 1a). Along the *c* axis, these moieties are distributed in two alternating layers, from which the aromatic rings of a specific layer are off-set from those of the layers directly above and below, avoiding efficient π-π stacking (Fig. 1b). The two average planes containing the phenyl rings are mutually rotated by *ca.* 40°, which increases the distance between hydrogen atoms of neighbouring pb^-^ anions.

The hydrophilic layer, which is formed by the carboxylate groups, the [Cu(en)_2_(H_2_O)_2_]^2+^ cations and the water molecules of crystallisation, exhibits extensive crystallographic disorder. On the one hand, the centrosymmetric cation has two possible mutually-tilted crystallographic positions (rates of occupancy of 1/3 and 2/3; see Fig. 2), which have in common the two carbon atoms and the Cu centre. For each possibility the Cu centre exhibits a typical octahedral coordination environment with a strong Jahn-Teller distortion: the Cu—N bonds (equatorial planes) range from 2.006 (2) to 2.035 (4) Å, and the Cu—O_water_ (apical positions) are either 2.601 (2) Å (2/3 occupancy) or 2.495 (4) Å (1/3 occupancy); however, the *cis* octahedral angles fall within a rather short range around the ideal value: 84.65 (15) – 95.35 (15) ° (Table 1).

The crystal structure is rich with a variety of hydrogen bonds due to the presence of amines, carboxylates and water molecules (both coordinated and uncoordinated). The formed hydrogen bonding sub-network is strongly affected by the aforementioned crystal disorder plus some additional disorder associated with the two uncoordinated water molecules (O2W and O4W). The coordinated O3W and uncoordinated O2W water molecules, plus the O1 oxygen atom of the carboxylate group are engaged in a series of strong [O···O distances ranging from 2.668 (2) to 2.932 (3) Å; Table 2] and rather directional [angles in the range 156 (5) – 168 (5) °; Table 2] O—H···O hydrogen bonds, forming an almost planar supramolecular hexagon (longest distance to the average plane of *ca.* 0.17 Å) with a graph set motif R_6_^4^(12) (Grell *et al.*, 1999) (Fig. 3). Remarkably, the carboxylate group further establishes bridges between adjacent supramolecular hexagons [O2W—H2M···O2 at 1.731 (10) Å; Fig. 3], leading to a 1-D hydrogen bonded chain running parallel the *a* axis (Fig. 3). The amino groups also participate in the hydrogen bonding network with rather directional interactions [angles are in the range 141 – 174 °; Table 2], even though the N···O distances with the O2 carboxylate atom and the water molecules are relatively long [N1···O2 2.986 (3) Å, N2···O2 3.037 (3) Å and N1···O2W 3.093 (3) Å].

### Experimental

All chemicals were purchased from commercial sources and were used as received without any further purification. An aqueous solution of Cu(NO_3_)_2_^.^3H_2_O (Sigma-Aldrich, 0.80 g, 3.3 mmol, 5 ml) was added to a solution of ethylenediamine (Riedel-de Haën, 0.5 ml, 7.3 mmol) in water (5 ml). After 1 min, a deep-violet solution was formed. Slow evaporation of the aqueous solution yielded crystalline plates of [Cu(en)_2_(H_2_O)_2_](NO_3_)_2_ after 15 days.

An aqueous solution of KOH (Eka, 0.106 g, 1.89 mmol, 10 ml) was added to a suspension of 4-phenylbenzoic acid (Hpb, Sigma-Aldrich, 0.375 g, 0.945 mmol) in water (40 ml). The resulting solution was filtered and added to an aqueous solution of [Cu(en)_2_(H_2_O)_2_](NO_3_)_2_ (0.228 g, 0.945 mmol, 10 ml). The precipitation of a blue-violet powder was immediate. The slow evaporation of the aqueous solution yielded crystalline needles of the title complex after 60 days.

Selected FT—IR (KBr, cm^-1^): ν(NH_2_) = 3363*s*, 3307*s*, 3216*s* and 3138*m*; ν_asym_(—COO^-^) = 1596*vs*, 1541*vs* and 1577*vs*; 1447*m*; ν_sym_(—COO^-^) = 1395*vs*; 1101*m*; 1045*s*; γ(-CH, di-subs. Ph) = 838*m* and 800*m*; γ(-CH, mono-subs. Ph) = 751*s*; 694*m*; 525*m*; 461*m*.

### Refinement

Hydrogen atoms bound to carbon and nitrogen were located at their idealized positions and were included in the final structural model in riding-motion approximation with: C—H = 0.95 Å (aromatic) and 0.95 Å (—CH_2_ moieties); N—H = 0.92 Å. The isotropic thermal displacement parameters for these atoms were fixed at 1.2 times *U*_eq_ of the respective parent atom.

H atoms associated with the four crystallographically independent water molecules were directly located from difference Fourier maps and included in the structure with the O—H and H···H distances restrained to 0.95 (1) and 1.55 (1) Å, respectively, in order to ensure a chemically reasonable geometry for these moieties. The *U*_iso_ of these H-atoms were fixed at 1.5 times *U*_eq_ of the parent O-atoms.

The crystallographically independent ethylendiamine moiety was found to be disordered over two distinct positions with rates of occupancy of 2/3 and 1/3, respectively (calculated from unrestrained refinements for the respective sites occupancies). The analogous pairs of carbon atoms of these moieties (C1 and C1'; C2 and C2') were located at the same crystallographic positions and were further included in the final structural model with identical anisotropic displacement parameters.

### Crystal data

**Table:**

[Cu(C_2_H_8_N_2_)_2_(H_2_O)_2_](C_13_H_9_O_2_)_2_·2.66H_2_O	*F*(000) = 699
*M**_r_* = 662.20	*D*_x_ = 1.406 Mg m^−^^3^
Monoclinic, *P*2_1_/*n*	Mo *K*α radiation, λ = 0.71073 Å
Hall symbol: -P 2yn	Cell parameters from 9591 reflections
*a* = 6.1466 (6) Å	θ = 3.3–30.5°
*b* = 34.984 (3) Å	µ = 0.76 mm^−^^1^
*c* = 7.3101 (7) Å	*T* = 150 K
β = 95.819 (4)°	Needle, violet
*V* = 1563.8 (3) Å^3^	0.13 × 0.10 × 0.06 mm
*Z* = 2	

### Data collection

**Table:**

Bruker X8 Kappa CCD APEXII diffractometer	4736 independent reflections
Radiation source: fine-focus sealed tube	3908 reflections with *I* > 2σ(*I*)
graphite	*R*_int_ = 0.034
ω/φ scans	θ_max_ = 30.5°, θ_min_ = 3.6°
Absorption correction: multi-scan (*SADABS*; Sheldrick, 1997)	*h* = −8→8
*T*_min_ = 0.908, *T*_max_ = 0.956	*k* = −47→49
26073 measured reflections	*l* = −10→9

### Refinement

**Table:**

Refinement on *F*^2^	Primary atom site location: structure-invariant direct methods
Least-squares matrix: full	Secondary atom site location: difference Fourier map
*R*[*F*^2^ > 2σ(*F*^2^)] = 0.041	Hydrogen site location: inferred from neighbouring sites
*wR*(*F*^2^) = 0.137	H atoms treated by a mixture of independent and constrained refinement
*S* = 1.10	*w* = 1/[σ^2^(*F*_o_^2^) + (0.0755*P*)^2^ + 0.5782*P*] where *P* = (*F*_o_^2^ + 2*F*_c_^2^)/3
4736 reflections	(Δ/σ)_max_ < 0.001
259 parameters	Δρ_max_ = 0.52 e Å^−^^3^
15 restraints	Δρ_min_ = −0.66 e Å^−^^3^

### Special details

**Table:**

Geometry. All esds (except the esd in the dihedral angle between two l.s. planes) are estimated using the full covariance matrix. The cell esds are taken into account individually in the estimation of esds in distances, angles and torsion angles; correlations between esds in cell parameters are only used when they are defined by crystal symmetry. An approximate (isotropic) treatment of cell esds is used for estimating esds involving l.s. planes.
Refinement. Refinement of *F*^2^ against ALL reflections. The weighted *R*-factor wR and goodness of fit *S* are based on *F*^2^, conventional *R*-factors *R* are based on *F*, with *F* set to zero for negative *F*^2^. The threshold expression of *F*^2^ > σ(*F*^2^) is used only for calculating *R*-factors(gt) etc. and is not relevant to the choice of reflections for refinement. *R*-factors based on *F*^2^ are statistically about twice as large as those based on *F*, and *R*- factors based on ALL data will be even larger.

### Fractional atomic coordinates and isotropic or equivalent isotropic displacement parameters (Å^2^)

**Table:**

	*x*	*y*	*z*	*U*_iso_*/*U*_eq_	Occ. (<1)
Cu1	0.5000	0.0000	0.0000	0.02840 (11)	
N1	0.6260 (4)	0.04498 (6)	0.1479 (3)	0.0285 (4)	0.67
H1A	0.5747	0.0453	0.2618	0.034*	0.67
H1B	0.7761	0.0434	0.1639	0.034*	0.67
N2	0.3249 (4)	0.03936 (6)	−0.1506 (3)	0.0326 (5)	0.67
H2A	0.3829	0.0428	−0.2607	0.039*	0.67
H2B	0.1831	0.0310	−0.1755	0.039*	0.67
C1	0.5555 (4)	0.08119 (5)	0.0418 (3)	0.0337 (4)	0.67
H1C	0.5564	0.1032	0.1272	0.040*	0.67
H1D	0.6583	0.0867	−0.0506	0.040*	0.67
C2	0.3274 (3)	0.07512 (5)	−0.0527 (3)	0.0336 (4)	0.67
H2C	0.2201	0.0744	0.0395	0.040*	0.67
H2D	0.2878	0.0963	−0.1393	0.040*	0.67
N1'	0.6818 (6)	0.04865 (12)	0.0030 (6)	0.0247 (8)	0.33
H1E	0.7995	0.0465	0.0907	0.030*	0.33
H1F	0.7345	0.0518	−0.1095	0.030*	0.33
N2'	0.2445 (6)	0.03581 (11)	−0.0017 (6)	0.0237 (8)	0.33
H2E	0.1326	0.0280	−0.0866	0.028*	0.33
H2F	0.1935	0.0365	0.1123	0.028*	0.33
C1'	0.5555 (4)	0.08119 (5)	0.0418 (3)	0.0337 (4)	0.33
H1G	0.6207	0.1047	−0.0051	0.040*	0.33
H1H	0.5507	0.0839	0.1762	0.040*	0.33
C2'	0.3274 (3)	0.07512 (5)	−0.0527 (3)	0.0336 (4)	0.33
H2G	0.3299	0.0769	−0.1876	0.040*	0.33
H2H	0.2282	0.0952	−0.0139	0.040*	0.33
O1W	0.4441 (9)	−0.00131 (10)	−0.3429 (6)	0.0369 (10)	0.33
H1M	0.467 (14)	0.0206 (7)	−0.415 (6)	0.055*	0.33
H1N	0.473 (14)	−0.0236 (7)	−0.410 (7)	0.055*	0.33
O1	0.3256 (2)	0.42163 (4)	0.1362 (2)	0.0358 (3)	
O2	−0.0141 (2)	0.42725 (4)	0.00400 (19)	0.0357 (3)	
C3	0.1443 (3)	0.40764 (5)	0.0747 (2)	0.0281 (3)	
C4	0.1163 (3)	0.36494 (5)	0.0880 (2)	0.0246 (3)	
C5	0.2925 (3)	0.34223 (5)	0.1570 (2)	0.0279 (3)	
H5	0.4286	0.3538	0.1973	0.034*	
C6	0.2705 (3)	0.30293 (5)	0.1670 (2)	0.0281 (3)	
H6	0.3920	0.2879	0.2144	0.034*	
C7	0.0717 (3)	0.28509 (4)	0.1083 (2)	0.0236 (3)	
C8	−0.1055 (3)	0.30806 (5)	0.0421 (2)	0.0265 (3)	
H8	−0.2425	0.2965	0.0035	0.032*	
C9	−0.0835 (3)	0.34750 (5)	0.0321 (2)	0.0272 (3)	
H9	−0.2052	0.3627	−0.0132	0.033*	
C10	0.0507 (3)	0.24284 (4)	0.1143 (2)	0.0245 (3)	
C11	0.2192 (3)	0.21931 (5)	0.0648 (3)	0.0288 (3)	
H11	0.3483	0.2304	0.0270	0.035*	
C12	0.1993 (3)	0.17987 (5)	0.0705 (3)	0.0343 (4)	
H12	0.3136	0.1642	0.0343	0.041*	
C13	0.0136 (3)	0.16311 (5)	0.1287 (3)	0.0353 (4)	
H13	0.0019	0.1361	0.1346	0.042*	
C14	−0.1549 (3)	0.18613 (5)	0.1784 (3)	0.0325 (4)	
H14	−0.2825	0.1748	0.2182	0.039*	
C15	−0.1371 (3)	0.22569 (5)	0.1697 (2)	0.0283 (3)	
H15	−0.2541	0.2412	0.2019	0.034*	
O2W	0.5759 (3)	0.43249 (6)	0.8469 (2)	0.0544 (5)	
H2M	0.716 (3)	0.4273 (15)	0.908 (4)	0.082*	0.67
H2N	0.474 (4)	0.4355 (17)	0.935 (3)	0.082*	0.67
H2O	0.563 (15)	0.4550 (11)	0.918 (8)	0.082*	0.33
H2P	0.510 (14)	0.4117 (13)	0.904 (9)	0.082*	0.33
O3W	0.1809 (3)	−0.00801 (6)	0.2058 (3)	0.0345 (4)	0.67
H3M	0.124 (6)	0.0139 (5)	0.261 (4)	0.052*	0.67
H3N	0.184 (6)	−0.0289 (5)	0.289 (3)	0.052*	0.67
O4W	0.0081 (8)	0.99783 (6)	0.3679 (12)	0.067 (2)	0.33
H4M	0.0656	0.9728	0.3644	0.101*	0.33
H4N	0.1242 (18)	1.01587 (19)	0.377 (17)	0.101*	0.33

### Atomic displacement parameters (Å^2^)

**Table:**

	*U*^11^	*U*^22^	*U*^33^	*U*^12^	*U*^13^	*U*^23^
Cu1	0.03755 (18)	0.01744 (15)	0.02768 (18)	0.00265 (10)	−0.00905 (12)	−0.00195 (10)
N1	0.0365 (11)	0.0218 (9)	0.0259 (10)	−0.0002 (8)	−0.0038 (8)	−0.0054 (8)
N2	0.0453 (13)	0.0211 (10)	0.0288 (11)	0.0044 (9)	−0.0085 (9)	−0.0006 (8)
C1	0.0507 (10)	0.0214 (8)	0.0297 (8)	−0.0054 (7)	0.0080 (7)	−0.0036 (6)
C2	0.0462 (10)	0.0230 (8)	0.0325 (9)	0.0091 (7)	0.0081 (8)	0.0035 (7)
N1'	0.0226 (17)	0.027 (2)	0.0234 (19)	−0.0036 (14)	−0.0015 (14)	0.0001 (15)
N2'	0.0203 (17)	0.0252 (18)	0.0253 (19)	−0.0006 (14)	0.0018 (14)	−0.0003 (15)
C1'	0.0507 (10)	0.0214 (8)	0.0297 (8)	−0.0054 (7)	0.0080 (7)	−0.0036 (6)
C2'	0.0462 (10)	0.0230 (8)	0.0325 (9)	0.0091 (7)	0.0081 (8)	0.0035 (7)
O1W	0.065 (3)	0.0230 (18)	0.0216 (18)	−0.0027 (16)	−0.0006 (18)	0.0007 (13)
O1	0.0410 (7)	0.0305 (6)	0.0368 (7)	−0.0123 (5)	0.0081 (6)	−0.0007 (5)
O2	0.0386 (7)	0.0244 (6)	0.0462 (9)	0.0002 (5)	0.0140 (6)	0.0015 (5)
C3	0.0374 (8)	0.0246 (7)	0.0247 (8)	−0.0056 (6)	0.0143 (6)	−0.0024 (6)
C4	0.0303 (7)	0.0227 (7)	0.0219 (7)	−0.0030 (6)	0.0076 (6)	−0.0022 (6)
C5	0.0277 (7)	0.0292 (8)	0.0267 (8)	−0.0051 (6)	0.0015 (6)	−0.0020 (6)
C6	0.0259 (7)	0.0298 (8)	0.0277 (8)	0.0004 (6)	−0.0014 (6)	−0.0001 (6)
C7	0.0263 (7)	0.0232 (7)	0.0211 (7)	−0.0008 (5)	0.0020 (5)	−0.0001 (6)
C8	0.0256 (7)	0.0236 (7)	0.0297 (8)	−0.0031 (6)	0.0004 (6)	0.0004 (6)
C9	0.0278 (7)	0.0245 (7)	0.0293 (8)	0.0001 (6)	0.0036 (6)	0.0009 (6)
C10	0.0291 (7)	0.0233 (7)	0.0203 (7)	−0.0007 (5)	−0.0016 (5)	0.0013 (5)
C11	0.0287 (7)	0.0263 (8)	0.0307 (8)	0.0026 (6)	−0.0006 (6)	0.0022 (6)
C12	0.0352 (9)	0.0266 (8)	0.0392 (10)	0.0074 (6)	−0.0048 (7)	−0.0010 (7)
C13	0.0442 (10)	0.0226 (8)	0.0368 (10)	−0.0002 (7)	−0.0076 (8)	0.0028 (7)
C14	0.0390 (9)	0.0274 (8)	0.0304 (9)	−0.0064 (7)	0.0002 (7)	0.0028 (7)
C15	0.0319 (8)	0.0268 (8)	0.0260 (8)	−0.0016 (6)	0.0028 (6)	−0.0008 (6)
O2W	0.0487 (9)	0.0846 (13)	0.0313 (8)	−0.0280 (9)	0.0115 (6)	−0.0046 (8)
O3W	0.0367 (10)	0.0221 (8)	0.0430 (12)	0.0042 (7)	−0.0037 (9)	−0.0011 (8)
O4W	0.040 (3)	0.028 (2)	0.129 (7)	−0.0058 (17)	−0.009 (3)	0.004 (3)

### Geometric parameters (Å, °)

**Table:**

Cu1—N1	2.019 (2)	C3—C4	1.508 (2)
Cu1—N1'	2.035 (4)	C4—C9	1.395 (2)
Cu1—N2	2.006 (2)	C4—C5	1.395 (2)
Cu1—N2'	2.008 (4)	C5—C6	1.384 (2)
Cu1—O1W	2.496 (4)	C5—H5	0.9500
Cu1—O3W	2.605 (2)	C6—C7	1.400 (2)
Cu1—N2^i^	2.006 (2)	C6—H6	0.9500
Cu1—N2'^i^	2.008 (4)	C7—C8	1.400 (2)
Cu1—N1^i^	2.019 (2)	C7—C10	1.485 (2)
Cu1—N1'^i^	2.035 (4)	C8—C9	1.389 (2)
Cu1—O1W^i^	2.496 (4)	C8—H8	0.9500
Cu1—O3W^i^	2.605 (2)	C9—H9	0.9500
N1—C1	1.525 (3)	C10—C15	1.397 (2)
N1—H1A	0.9200	C10—C11	1.399 (2)
N1—H1B	0.9200	C11—C12	1.386 (2)
N2—C2	1.441 (3)	C11—H11	0.9500
N2—H2A	0.9200	C12—C13	1.388 (3)
N2—H2B	0.9200	C12—H12	0.9500
C1—C2	1.514 (3)	C13—C14	1.389 (3)
C1—H1C	0.9900	C13—H13	0.9500
C1—H1D	0.9900	C14—C15	1.390 (2)
C2—H2C	0.9900	C14—H14	0.9500
C2—H2D	0.9900	C15—H15	0.9500
N1'—H1E	0.9200	O2W—H2M	0.9500 (11)
N1'—H1F	0.9200	O2W—H2N	0.9500 (10)
N2'—H2E	0.9200	O2W—H2O	0.9500 (11)
N2'—H2F	0.9200	O2W—H2P	0.9500 (12)
O1W—H1M	0.9500 (10)	O3W—H3M	0.9499 (10)
O1W—H1N	0.9500 (10)	O3W—H3N	0.9500 (10)
O1—C3	1.258 (2)	O4W—H4M	0.9457
O2—C3	1.258 (2)	O4W—H4N	0.950 (2)
			
N2—Cu1—N1	85.08 (9)	H2A—N2—H2B	108.1
N2—Cu1—N1^i^	94.92 (9)	C2—C1—N1	108.52 (15)
N2'—Cu1—N1'	84.65 (15)	C2—C1—Cu1	73.63 (9)
N2'—Cu1—N1'^i^	95.35 (15)	C2—C1—H1C	110.0
N1'—Cu1—O1W	92.62 (15)	N1—C1—H1C	110.0
N1'—Cu1—O1W^i^	87.38 (15)	Cu1—C1—H1C	145.9
N2^i^—Cu1—N2	180.00 (9)	C2—C1—H1D	110.0
N2^i^—Cu1—N2'^i^	36.18 (14)	N1—C1—H1D	110.0
N2—Cu1—N2'^i^	143.82 (14)	Cu1—C1—H1D	101.4
N2^i^—Cu1—N2'	143.82 (14)	H1C—C1—H1D	108.4
N2—Cu1—N2'	36.18 (14)	N2—C2—C1	108.13 (16)
N2'^i^—Cu1—N2'	180.0 (3)	C1—C2—Cu1	75.67 (9)
N2^i^—Cu1—N1^i^	85.08 (9)	N2—C2—H2C	110.1
N2'^i^—Cu1—N1^i^	76.97 (13)	C1—C2—H2C	110.1
N2'—Cu1—N1^i^	103.03 (13)	Cu1—C2—H2C	98.7
N2^i^—Cu1—N1	94.92 (9)	N2—C2—H2D	110.1
N2'^i^—Cu1—N1	103.03 (13)	C1—C2—H2D	110.1
N2'—Cu1—N1	76.97 (13)	Cu1—C2—H2D	147.5
N1^i^—Cu1—N1	180.00 (14)	H2C—C2—H2D	108.4
N2^i^—Cu1—N1'^i^	72.26 (13)	Cu1—N1'—H1E	109.4
N2—Cu1—N1'^i^	107.74 (13)	Cu1—N1'—H1F	109.4
N2'^i^—Cu1—N1'^i^	84.65 (15)	H1E—N1'—H1F	108.0
N1^i^—Cu1—N1'^i^	33.06 (14)	Cu1—N2'—H2E	110.4
N1—Cu1—N1'^i^	146.94 (14)	Cu1—N2'—H2F	110.4
N2^i^—Cu1—N1'	107.74 (13)	H2E—N2'—H2F	108.6
N2—Cu1—N1'	72.26 (13)	Cu1—O1W—H1M	122 (3)
N2'^i^—Cu1—N1'	95.35 (15)	Cu1—O1W—H1N	121 (4)
N1^i^—Cu1—N1'	146.94 (14)	H1M—O1W—H1N	109.34 (16)
N1—Cu1—N1'	33.06 (14)	O2—C3—O1	123.74 (16)
N1'^i^—Cu1—N1'	180.00 (13)	O2—C3—C4	118.56 (15)
N2^i^—Cu1—O1W^i^	56.63 (12)	O1—C3—C4	117.70 (16)
N2—Cu1—O1W^i^	123.37 (12)	C9—C4—C5	119.00 (15)
N2'^i^—Cu1—O1W^i^	88.69 (17)	C9—C4—C3	121.13 (15)
N2'—Cu1—O1W^i^	91.31 (17)	C5—C4—C3	119.86 (15)
N1^i^—Cu1—O1W^i^	123.96 (11)	C6—C5—C4	120.56 (15)
N1—Cu1—O1W^i^	56.04 (11)	C6—C5—H5	119.7
N1'^i^—Cu1—O1W^i^	92.62 (15)	C4—C5—H5	119.7
N2^i^—Cu1—O1W	123.37 (12)	C5—C6—C7	120.87 (15)
N2—Cu1—O1W	56.63 (12)	C5—C6—H6	119.6
N2'^i^—Cu1—O1W	91.31 (17)	C7—C6—H6	119.6
N2'—Cu1—O1W	88.69 (17)	C8—C7—C6	118.33 (15)
N1^i^—Cu1—O1W	56.04 (11)	C8—C7—C10	120.99 (14)
N1—Cu1—O1W	123.96 (11)	C6—C7—C10	120.68 (14)
N1'^i^—Cu1—O1W	87.38 (15)	C9—C8—C7	120.77 (15)
O1W^i^—Cu1—O1W	180.0	C9—C8—H8	119.6
N2^i^—Cu1—O3W	90.15 (9)	C7—C8—H8	119.6
N2—Cu1—O3W	89.85 (9)	C8—C9—C4	120.45 (15)
N2'^i^—Cu1—O3W	124.30 (13)	C8—C9—H9	119.8
N2'—Cu1—O3W	55.70 (13)	C4—C9—H9	119.8
N1^i^—Cu1—O3W	87.42 (8)	C15—C10—C11	118.51 (15)
N1—Cu1—O3W	92.58 (8)	C15—C10—C7	120.86 (15)
N1'^i^—Cu1—O3W	58.09 (13)	C11—C10—C7	120.64 (15)
N1'—Cu1—O3W	121.91 (13)	C12—C11—C10	120.55 (17)
O1W^i^—Cu1—O3W	57.13 (13)	C12—C11—H11	119.7
O1W—Cu1—O3W	122.87 (13)	C10—C11—H11	119.7
N2^i^—Cu1—O3W^i^	89.85 (9)	C11—C12—C13	120.49 (17)
N2—Cu1—O3W^i^	90.15 (9)	C11—C12—H12	119.8
N2'^i^—Cu1—O3W^i^	55.70 (13)	C13—C12—H12	119.8
N2'—Cu1—O3W^i^	124.30 (13)	C12—C13—C14	119.58 (16)
N1^i^—Cu1—O3W^i^	92.58 (8)	C12—C13—H13	120.2
N1—Cu1—O3W^i^	87.42 (8)	C14—C13—H13	120.2
N1'^i^—Cu1—O3W^i^	121.91 (13)	C13—C14—C15	120.06 (17)
N1'—Cu1—O3W^i^	58.09 (13)	C13—C14—H14	120.0
O1W^i^—Cu1—O3W^i^	122.87 (13)	C15—C14—H14	120.0
O1W—Cu1—O3W^i^	57.13 (13)	C14—C15—C10	120.80 (16)
O3W—Cu1—O3W^i^	180.00 (5)	C14—C15—H15	119.6
C1—N1—Cu1	107.53 (14)	C10—C15—H15	119.6
C1—N1—H1A	110.2	H2M—O2W—H2N	109.33 (16)
Cu1—N1—H1A	110.2	H2M—O2W—H2O	91 (6)
C1—N1—H1B	110.2	H2N—O2W—H2O	56 (4)
Cu1—N1—H1B	110.2	H2M—O2W—H2P	93 (6)
H1A—N1—H1B	108.5	H2N—O2W—H2P	56 (4)
C2—N2—Cu1	110.19 (15)	H2O—O2W—H2P	109.33 (17)
C2—N2—H2A	109.6	Cu1—O3W—H3M	119 (2)
Cu1—N2—H2A	109.6	Cu1—O3W—H3N	119 (2)
C2—N2—H2B	109.6	H3M—O3W—H3N	109.33 (16)
Cu1—N2—H2B	109.6	H4M—O4W—H4N	109.7
			
N2^i^—Cu1—N1—C1	168.87 (15)	N2'^i^—Cu1—C2—N2	102.4 (3)
N2—Cu1—N1—C1	−11.13 (15)	N2'—Cu1—C2—N2	−77.6 (3)
N2'^i^—Cu1—N1—C1	133.12 (18)	N1^i^—Cu1—C2—N2	−19.2 (2)
N2'—Cu1—N1—C1	−46.88 (18)	N1—Cu1—C2—N2	160.8 (2)
N1'^i^—Cu1—N1—C1	−126.5 (2)	N1'^i^—Cu1—C2—N2	−59.0 (2)
N1'—Cu1—N1—C1	53.5 (2)	N1'—Cu1—C2—N2	121.0 (2)
O1W^i^—Cu1—N1—C1	−147.5 (2)	O1W^i^—Cu1—C2—N2	−157.5 (2)
O1W—Cu1—N1—C1	32.5 (2)	O1W—Cu1—C2—N2	22.5 (2)
O3W—Cu1—N1—C1	−100.76 (15)	O3W—Cu1—C2—N2	−102.02 (19)
O3W^i^—Cu1—N1—C1	79.24 (15)	O3W^i^—Cu1—C2—N2	77.98 (19)
N2'^i^—Cu1—N2—C2	−121.7 (2)	N2^i^—Cu1—C2—C1	39.2 (2)
N2'—Cu1—N2—C2	58.3 (2)	N2—Cu1—C2—C1	−140.8 (2)
N1^i^—Cu1—N2—C2	163.71 (18)	N2'^i^—Cu1—C2—C1	−38.4 (3)
N1—Cu1—N2—C2	−16.29 (18)	N2'—Cu1—C2—C1	141.6 (3)
N1'^i^—Cu1—N2—C2	132.5 (2)	N1^i^—Cu1—C2—C1	−160.01 (12)
N1'—Cu1—N2—C2	−47.5 (2)	N1—Cu1—C2—C1	19.99 (12)
O1W^i^—Cu1—N2—C2	27.0 (3)	N1'^i^—Cu1—C2—C1	160.24 (18)
O1W—Cu1—N2—C2	−153.0 (3)	N1'—Cu1—C2—C1	−19.76 (18)
O3W—Cu1—N2—C2	76.31 (18)	O1W^i^—Cu1—C2—C1	61.70 (16)
O3W^i^—Cu1—N2—C2	−103.69 (18)	O1W—Cu1—C2—C1	−118.30 (16)
Cu1—N1—C1—C2	35.43 (19)	O3W—Cu1—C2—C1	117.21 (12)
N2^i^—Cu1—C1—C2	−158.57 (13)	O3W^i^—Cu1—C2—C1	−62.79 (12)
N2—Cu1—C1—C2	21.43 (13)	O2—C3—C4—C9	−3.1 (2)
N2'^i^—Cu1—C1—C2	157.87 (17)	O1—C3—C4—C9	176.50 (16)
N2'—Cu1—C1—C2	−22.13 (17)	O2—C3—C4—C5	176.68 (16)
N1^i^—Cu1—C1—C2	35.0 (2)	O1—C3—C4—C5	−3.7 (2)
N1—Cu1—C1—C2	−145.0 (2)	C9—C4—C5—C6	1.0 (3)
N1'^i^—Cu1—C1—C2	−36.8 (3)	C3—C4—C5—C6	−178.83 (15)
N1'—Cu1—C1—C2	143.2 (3)	C4—C5—C6—C7	0.0 (3)
O1W^i^—Cu1—C1—C2	−118.33 (16)	C5—C6—C7—C8	−1.1 (3)
O1W—Cu1—C1—C2	61.67 (16)	C5—C6—C7—C10	178.21 (16)
O3W—Cu1—C1—C2	−63.05 (12)	C6—C7—C8—C9	1.0 (3)
O3W^i^—Cu1—C1—C2	116.95 (12)	C10—C7—C8—C9	−178.23 (16)
N2^i^—Cu1—C1—N1	−13.52 (19)	C7—C8—C9—C4	0.0 (3)
N2—Cu1—C1—N1	166.48 (19)	C5—C4—C9—C8	−1.0 (3)
N2'^i^—Cu1—C1—N1	−57.1 (2)	C3—C4—C9—C8	178.81 (16)
N2'—Cu1—C1—N1	122.9 (2)	C8—C7—C10—C15	−41.2 (2)
N1^i^—Cu1—C1—N1	180.0	C6—C7—C10—C15	139.60 (17)
N1'^i^—Cu1—C1—N1	108.2 (3)	C8—C7—C10—C11	138.95 (17)
N1'—Cu1—C1—N1	−71.8 (3)	C6—C7—C10—C11	−40.3 (2)
O1W^i^—Cu1—C1—N1	26.7 (2)	C15—C10—C11—C12	0.1 (2)
O1W—Cu1—C1—N1	−153.3 (2)	C7—C10—C11—C12	−179.99 (16)
O3W—Cu1—C1—N1	81.99 (16)	C10—C11—C12—C13	−1.2 (3)
O3W^i^—Cu1—C1—N1	−98.01 (16)	C11—C12—C13—C14	1.2 (3)
Cu1—N2—C2—C1	40.1 (2)	C12—C13—C14—C15	0.0 (3)
N1—C1—C2—N2	−50.0 (2)	C13—C14—C15—C10	−1.1 (3)
Cu1—C1—C2—N2	−26.13 (15)	C11—C10—C15—C14	1.0 (2)
N1—C1—C2—Cu1	−23.88 (13)	C7—C10—C15—C14	−178.88 (16)
N2^i^—Cu1—C2—N2	180.0		

Symmetry codes: (i) −*x*+1, −*y*, −*z*.

### Hydrogen-bond geometry (Å, °)

**Table:**

*D*—H···*A*	*D*—H	H···*A*	*D*···*A*	*D*—H···*A*
N1—H1A···O2^ii^	0.92	2.13	2.986 (3)	154
N1—H1B···O2W^iii^	0.92	2.32	3.093 (3)	141
N2—H2A···O2^iii^	0.92	2.16	3.037 (3)	158
N1'—H1E···O2W^iii^	0.92	2.51	3.375 (4)	157
N1'—H1F···O1^iii^	0.92	2.20	3.087 (5)	161
N2'—H2E···O4W^iv^	0.92	2.33	3.181 (9)	154
N2'—H2F···O2W^v^	0.92	2.21	3.055 (5)	152
O1W—H1M···O2^iii^	0.95 (1)	1.92 (1)	2.844 (4)	163 (4)
O1W—H1N···O2^vi^	0.95 (1)	1.88 (1)	2.814 (4)	169 (4)
O2W—H2M···O2^vii^	0.95 (1)	1.73 (1)	2.668 (2)	168 (5)
O2W—H2N···O1^viii^	0.95 (1)	1.87 (2)	2.765 (2)	156 (5)
O3W—H3M···O2W^v^	0.95 (1)	2.01 (1)	2.932 (3)	163 (3)
O3W—H3N···O1^ix^	0.95 (1)	1.82 (1)	2.721 (3)	158 (3)
O4W—H4M···O1^x^	0.95 (1)	1.91	2.8562 (17)	178

Symmetry codes: (ii) *x*+1/2, −*y*+1/2, *z*+1/2; (iii) *x*+1/2, −*y*+1/2, *z*−1/2; (iv) −*x*, −*y*+1, −*z*; (v) *x*−1/2, −*y*+1/2, *z*−1/2; (vi) −*x*+1/2, *y*−1/2, −*z*−1/2; (vii) *x*+1, *y*, *z*+1; (viii) *x*, *y*, *z*+1; (ix) −*x*+1/2, *y*−1/2, −*z*+1/2; (x) −*x*+1/2, *y*+1/2, −*z*+1/2.
